# Case Report: Venoarterial Extracorporeal Membrane Oxygenation Support for Caowu-Induced Cardiac Arrest

**DOI:** 10.3389/fmed.2021.731163

**Published:** 2021-11-03

**Authors:** Binbin Ren, Liming Wang, Kun Chen, Lin Chen, Huabin Wang

**Affiliations:** ^1^Department of Infectious Disease, School of Medicine, Affiliated Jinhua Hospital, Zhejiang University, Jinhua, China; ^2^Department of Intensive Care Unit, School of Medicine, Affiliated Jinhua Hospital, Zhejiang University, Jinhua, China; ^3^Central Laboratory, Affiliated Jinhua Hospital, Zhejiang University School of Medicine, Jinhua, China

**Keywords:** VA-ECMO, aconitine, heart arrest, malignant arrhythmias, Caowu

## Abstract

**Introduction:** Caowu, the main root of the *Aconitum* plant, is widely used in China. Aconitine is the main toxic component of *Aconitum*, which can cause a variety of malignant arrhythmias and lead to death. Four patients who developed malignant arrhythmia after drinking medicinal wine containing Caowu were reported in this study. Cardiac arrest occurred soon after symptom onset. All patients received venoarterial extracorporeal membrane oxygenation (VA-ECMO) support after conservative medical treatment had failed. Patients who were directly transferred to our hospital received VA-ECMO support earlier than patients who were first treated at a local hospital. One patient received hemoperfusion in the emergency room before VA-ECMO support; the other three patients began hemoperfusion after VA-ECMO treatment. Surviving patients who received VA-ECMO earlier after symptom onset showed no obvious neurological complications. The patient who received a longer cardiopulmonary resuscitation time but received hemoperfusion before VA-ECMO had mild neurological complications. The mortality rate was 25% (1 of 4 patients). Two patients had thrombotic complications in venous vessels.

**Conclusions:** Cardiogenic shock due to refractory ventricular tachycardia caused by aconitine is lethal. Conservative supportive treatment did not provide a short-term antiarrhythmic effect and the cardiogenic shock was not well controlled. VA-ECMO treatment combined with hemoperfusion is promising temporary support to successfully treat aconitine-induced cardiogenic shock caused by refractory ventricular tachycardia.

## Introduction

*Aconitum* is a widely used Chinese herbal medicine. *Caowu* is the main root of the *Aconitum* plant and contains aconitine, a toxic component. Such herbs are either used as medicine or added to food or wine and are popular in China. However, the aconitine therapeutic dose is close to the poisonous or lethal dose. Thus, the main adverse effects are aconitine-induced ventricular tachyarrhythmia and heart arrest, which are also the leading causes of death (1). Patients with Caowu poisoning often present with a stubborn arrhythmia with poor response to conventional treatment, including many antiarrhythmic drugs, defibrillation, and cardioversion. Malignant arrhythmias occur repeatedly with unstable hemodynamics, which are difficult to control with conventional treatment.

Extracorporeal membrane oxygenation (ECMO) or extracorporeal life support to manage patients with drug-induced cardiogenic shock (DCS) have been increasingly reported (2, 3). However, there are no reports on ECMO treatment of patients with Caowu poisoning. Even reports of experiences of ECMO support of patients poisoned by Chinese herbal medicine are limited. However, patients can rapidly improve if the herbal medicine can be quickly cleared or the patients are provided temporary support to allow time for the elimination of the toxic substances. Thus, temporary life support for such patients is critical.

The two types of ECMO, venovenous ECMO (VV-ECMO) and venoarterial ECMOextracorporeal membrane oxygenation (VA-ECMO), were initially developed in the 1950s (4). VV-ECMO mainly provides adequate oxygenation to support respiratory failure, while VA-ECMO can provide both circulation and oxygenation support but is mainly used for circulation failure. VA-ECMO is increasingly used as an effective treatment for refractory cardiogenic shock, a bridge to heart transplantation, and myocardial or durable mechanical circulatory dysfunction (5). Cardiac dysfunction due to drug poisoning is usually temporary and reversible. Once the drug is cleared, the function of the heart would recover. VA-ECMO is the temporary life support which can provide mechanical support for circulation to gain time for its recovery caused by drug poisoning. To our knowledge, this is the first report of the use of ECMO to support circulation in cases of Caowu poisoning. We share our experiences about the treatment of such patients and also suggest that VA-ECMO treatment combined with hemoperfusion might be effective approaches to save the lives of these patients.

## Case Descriptions

Four patients aged from 46 to 55 years old experienced vomiting and numbness after drinking a medicinal liquor containing Caowu. All patients were male and healthy without diabetes, hypertension, coronary heart disease, or genetic disease. Two patients were sent to the local hospital, while two patients were sent to our hospital after symptom onset. When they arrived at the emergency room, they were conscious without obvious abnormalities in physical examination. Their lungs sounded clear, no wheezing and moist rales. Cardiac auscultation showed no obvious positive signs. No obvious tenderness or rebound pain were observed in the abdomen. The patients underwent immediate gastric lavage via nasogastric tubes. While in the emergency room, three patients began to lose consciousness; the other patient also deteriorated, with chest tightness and syncope. All four patients had ventricular arrhythmia and cardiac arrest at the onset of new symptoms. Conventional cardiopulmonary resuscitation was performed immediately. The patients also received mechanical ventilation and antiarrhythmic drugs, including lidocaine and amiodarone, which were administered intravenously after intravenous loading. Calcium gluconate and magnesium sulfate were administered to maintain the electrolyte balance, as well as an equilibrium solution and normal saline to ensure fluid resuscitation to correct cardiogenic shock with the use of norepinephrine at the same time. Electrical defibrillation was performed repeatedly, nevertheless, the cardiogenic shock of the patients did not improve even with high-dose of norepinephrine (>1ug/kg/min) administration. Gastric lavage was continued and cardiopulmonary resuscitation was performed. The sinus rhythm and circulation could not be maintained despite the prolonged cardiopulmonary resuscitation. Due to the lack of circulation improvement, the two patients in our hospital received VA-ECMO support. The local hospital where the other two patients were treated had no such device. Then our emergency response team traveled to the local hospital with our device and performed VA-ECMO for these two patients. The patients were then transferred to our hospital for further treatment. Patient 4 received hemoperfusion before VA-ECMO while the other three patients underwent hemoperfusion after VA-ECMO initiation. The hemoperfusion was performed 2 h each time, twice a day using the perfusion device—HA330-IIproduced by Jafron Biomedical Co., Ltd., China. All the patients still had ventricular arrhythmia repeatedly after receiving the ECMO support. However, when ventricular arrhythmia occurred, we did not perform the CPR or electrical defibrillation for the patients any more. Lidocaine and amiodarone were continued together with the support of the VA-ECMO and hemoperfusion to improve arrhythmia and circulation. The doses of norepinephrine gradually decreased with the improvement of circulation of the patients. Sinus rhythm became stable within 3–4 h after ECMO support, followed by sporadic ventricular premature. The heart function was monitored by bedside ultrasound, and the ECMO flow was adjusted to avoid left ventricular expansion. Unfractionated heparin maintained activated coagulation time around 180 s. The clinical characteristics and treatment information of the four patients are shown in Table 1. This study followed the tenets of the Declaration of Helsinki and was approved by the Ethics Committee of the Affiliated Jinhua Hospital, Zhejiang University School of Medicine.

**Table 1:** The clinical characteristics of the four patients.

	**Patient 1**	**Patient 2**	**Patient 3**	**Patient 4**
Gender	Male	Male	Male	Male
Age (years)	46	55	50	48
Symptoms	Vomiting, numbness, consciousness disorder	Vomiting, numbness, consciousness disorder	Vomiting, numbness, chest tightness, syncope	Vomiting, numbness, consciousness disorder
Time from drinking the medicinal liquor to symptom onset (h)	5.5	4.5	8	3
First treated place	Our hospital	Local hospital	Our hospital	Local hospital
Time from CPR to ECMO support (min)	45	80	50	90
Lactate (mmol/L)	9.9	5.5	4.6	8
PH	7.21	7.29	7.32	7.31
BE (mmol/L)	−8.3	−8.4	−9.2	−7.1
Limb ischemia complications	No	No	No	No
Bloodstream infection	No	No	No	No
Bleeding complications	No	No	No	No
Thrombotic complications	No	Yes	Yes	No
Time of norepinephrine use (h)	19	58	65	78
ECMO running time (h)	19	68	64	79
Hemoperfusion time (days)	<1	3	2	3
Time of mechanical ventilation (days)	<1	5	5	6
Left ventricular overload	No	No	No	No
NHISS scores on discharge	Die	4	0	2
Outcome	Die	Discharge	Discharge	Discharge

*ECMO, Extracorporeal Membrane Oxygenation; PH, Potential of Hydrogen; BE, Base Excess; NHISS, National Herb Study Society*.

## Results

The mean age of the four patients was 49.8 years. All four patients were male and received repeated traditional cardiopulmonary resuscitation, antiarrhythmic drugs including lidocaine (load capacity: 100 mg, maintained at 0.03 mg/kg after two repetitions), amiodarone (load capacity: 300 mg, maintained at 1 mg/min), gastric lavage, and fluid resuscitation. Noradrenaline (1–2 ug/kg/min) was used to maintain circulation but without significant improvement. The time elapsed from drinking the medicinal liquor to symptom onset ranged from 3 to 8 h. Patients 1 and 3 were first transported to our hospital for treatment. Patient 1 did not survive even when cardiac arrest occurred in the hospital and with VA-ECMO support. Patients 2 and 4 did not receive timely VA-ECMO because of a lack of equipment in the local hospital, and Patient 4 had a longer duration of cardiopulmonary resuscitation; however, his neurological complications (speech and sensation disorders) were milder compared with Patient 2 (dyspraxia, speech, and sensation disorders). Patient 3 survived without any obvious neurological complications. Two patients developed thrombotic complications. With the improved circulation, we removed ECMO when norepinephrine was discontinued or at low doses. Due to the poorer oxygenation (improved by conducting liquid management), the ECMO of Patient 2 was removed 10 h after the norepinephrine was discontinued. The VA-ECMO support times ranged from 64 to 79 h. The minimum follow-up time was more than half a year. Two patients received antithrombotic therapy for 3 months after discharge, and no further complications occurred. Neurological complications also improved in Patient 2 and Patient 4 (their NHISS scores were 3 and 1 after 3-month follow-up, respectively).

## Discussion

We first retrospectively reported the patients who developed malignant arrhythmia after drinking medicinal wine containing Caowu. Aconitum combined with alcohol will lead to more complex pathophysiology. We presented our experiences for rescuing patients with cardiogenic shock caused by drinking medicinal wine containing Caowu to help better manage such critical situation.

Due to the recoverability of heart rhythm in such patients, temporary life support for the patients with cardiogenic shock caused by toxicity is necessary. The methods for providing mechanical circulatory support for cardiogenic shock caused by drug poisoning include intra-aortic balloon counterpulsation (IABP), cardiopulmonary bypass, and ECMO. Among these, IABP (6) is the most commonly used; however, the limitation of this method is the requirement that the patient retain some heart function and an arterial systolic pressure above 40 mmHg. Moreover, IABP cannot supply oxygen. While cardiopulmonary bypass (7) can provide complete circulatory and respiratory support, it requires opening the chest cavity, a median incision, a venous cannula into the right atrium, and an arterial cannula into the ascending aorta, which make it difficult to provide quick and effective support for emergency conditions. VA-ECMO can provide complete cardiopulmonary support and is also convenient to perform, with fewer requirements for implementation (8). The use of VA-ECMO to support circulatory collapse caused by poisoning is increasingly reported. The first use of VA-ECMO to treat quinidine-induced poisoning was reported in 1997 (9). Lindsay et al. (3) systematically reviewed the treatment of DCS by VA-ECMO. A total of 104 patients were included, with a survival rate of 52.9%. VA-ECMO improved mean arterial pressure, systolic blood pressure, and diastolic blood pressure. The related metabolic indices and oxygenation were also significantly improved, thus demonstrating the significant advantages of VA-ECMO in the treatment of drug-induced cardiogenic shock. This study was the largest study of VA-ECMO to support patients with DCS.

The effectiveness and importance of Chinese herbal medicines have gradually been recognized. With the development of modern science and technology, Chinese herbal medicine has become more widely used worldwide. Caowu is a component of the *Aconitum* plant with many medicinal properties. A famous traditional Chinese medicine, Yunnan Baiyao, which is very popular in clinical diseases, contains Caowu (10). Caowu is also detoxified by the zygote (Terminalia chebula Retz) detox, and the Zhicao (Grass Aconitum) when it is prepared as part of NaRu-3 pills as a treatment for rheumatoid arthritis (11). There are many ways to process Caowu before developing it into medicine (11, 12). Due to the medicinal value of these Chinese herbal medicines since ancient times, Chinese folk remedies often soaked Chinese herbal medicines in wine, or added some herbal when cooking food. Poisoning incidents are also frequently reported (11, 13).

Aconitine is the most significant toxic component of *Aconitum* plants. It mainly acts on the heart and nervous system, causing a variety of arrhythmias, which may lead to death. The main mechanism of ethanol-induced toxicity is the inhibition of the central nervous system. However, the toxic effect of the combined action of aconitine and ethanol on the myocardium becomes even more complex. Studies on the mechanism of Caowu poisoning and Caowu combined with alcohol poisoning are increasing (14, 15); however, the mechanism requires further research.

Owing to its convenience and effectiveness, VA-ECMO has been increasingly used in patients with unstable circulation caused by various heart diseases. The value of VA-ECMO for cardiopulmonary resuscitation has also become increasingly prominent. However, acute poisoning is not a common reason for the use of VA-ECMO. Moreover, the use of VA-ECMO as a supportive treatment for Caowu-induced cardiac arrest has not been reported. There remains no effective detoxification therapy for Chinese herbal medicines such as Caowu. However, while ventricular arrhythmia in such patients may be stubborn, it is highly recoverable and may return to normal immediately after the drug is cleared. The essential treatment for such patients is timely life support to provide time for drug metabolism or to accelerate the drug clearance using methods such as the intermittent use of hemoperfusion. VA-ECMO provides sufficient circulatory support to provide more stable circulation. Once the ventricular arrhythmia stops, the patient's heart rhythm and circulation stabilize. Among the surviving patients in the present series, two developed neurological complications and two developed deep vein thrombosis. No left ventricular decompression was required. No severe bleeding events occurred during the VA-ECMO treatment. Nervous system complications and thrombotic events might be related to repeated refractory arrhythmia and long-duration cardiopulmonary resuscitation. One possible explanation for the death of Patient 1 might be an overdose of the medicinal liquor containing Caowu. As the patient showed no significant improvement, his family members decided to refuse further treatment. Patient 4 recovered with mild nervous system complications might be due to the earlier use of hemoperfusion, later combined with VA-ECMO support. The period of time between VA-ECMO and cardiopulmonary resuscitation in the patient in our study was longer than that recommended by the Extracorporeal Life Support Organization for patients with cardiac arrest due to various cardiac diseases (16). Part of the reason is that these patients can recover their heart rate after receiving traditional cardiopulmonary resuscitation, but sinus rhythm cannot be maintained. The potential benefits of the earlier use of VA-ECMO combined with hemoperfusion require additional clinical evidence.

In conclusion, the aconitine contained in Caowu can cause fatal arrhythmia and cardiac arrest. Effective cardiopulmonary resuscitation and VA-ECMO support combined with hemoperfusion could provide sufficient time for patients to recover. This is the first report on the effectiveness of VA-ECMO as a treatment in such emergency conditions. Our findings demonstrated its effectiveness, especially when combined with hemoperfusion. Nevertheless, when to perform VA-ECMO support would more benefit such patients requires further study.

## Data Availability Statement

The raw data supporting the conclusions of this article will be made available by the authors, without undue reservation.

## Ethics Statement

The studies involving human participants were reviewed and approved by Ethics Committee of the Affiliated Jinhua Hospital, Zhejiang University School of Medicine. The patients/participants provided their written informed consent to participate in this study.

## Author Contributions

LW, LC, and KC: study design. LC and BR: data collected. LC and HW: manuscript writing. All authors contributed to the article and approved the submitted version.

## Funding

This work was supported by the Grants: Key Research and Development Project of Zhejiang Province (2020C03019), and Youth Foundation of Jinhua Municipal Central Hospital (JY2020-2-10).

## Conflict of Interest

The authors declare that the research was conducted in the absence of any commercial or financial relationships that could be construed as a potential conflict of interest.

## Publisher's Note

All claims expressed in this article are solely those of the authors and do not necessarily represent those of their affiliated organizations, or those of the publisher, the editors and the reviewers. Any product that may be evaluated in this article, or claim that may be made by its manufacturer, is not guaranteed or endorsed by the publisher.
